# A Cross-Sectional Study on Attitudes to and Understanding of Risk of Acquisition of HIV: Design, Methods and Participant Characteristics

**DOI:** 10.2196/resprot.4873

**Published:** 2016-04-18

**Authors:** Janey Sewell, Andrew Speakman, Andrew N Phillips, Fiona C Lampe, Ada Miltz, Richard Gilson, David Asboe, Nneka Nwokolo, Christopher Scott, Sara Day, Martin Fisher, Amanda Clarke, Jane Anderson, Rebecca O'Connell, Vanessa Apea, Rageshri Dhairyawan, Mark Gompels, Paymaneh Farazmand, Sris Allan, Susan Mann, Jyoti Dhar, Alan Tang, S Tariq Sadiq, Stephen Taylor, Simon Collins, Lorraine Sherr, Graham Hart, Anne M Johnson, Alec Miners, Jonathan Elford, Alison Rodger

**Affiliations:** ^1^ Institute of Epidemiology and Health Care Research Department of Infection and Population Health UCL London United Kingdom; ^2^ Chelsea and Westminster NHS Foundation Trust London United Kingdom; ^3^ Brighton and Sussex University Hospitals NHS Trust Brighton United Kingdom; ^4^ Homerton University Hospital NHS Foundation Trust London United Kingdom; ^5^ Barts Health NHS Trust London United Kingdom; ^6^ Barking, Havering and Redbridge University Hospitals NHS Trust London United Kingdom; ^7^ North Bristol NHS Trust Bristol United Kingdom; ^8^ Calderdale and Huddersfield NHS Foundation Trust Huddersfield United Kingdom; ^9^ Coventry and Warwickshire Partnership NHS Trust Coventry United Kingdom; ^10^ King’s College Hospital NHS Foundation Trust London United Kingdom; ^11^ Staffordshire and Stoke on Trent Partnership NHS Trust Leicester United Kingdom; ^12^ Royal Berkshire NHS Foundation Trust Reading United Kingdom; ^13^ Institute for Infection and Immunity, St George's University of London London United Kingdom; ^14^ Heart of England NHS Foundation Trust Birmingham United Kingdom; ^15^ HIV i-Base London United Kingdom; ^16^ London School of Hygiene and Tropical Medicine London United Kingdom; ^17^ City University London London United Kingdom

**Keywords:** HIV infection, HIV negative, HIV undiagnosed, HIV transmission, HIV testing, men who have sex with men, black Africans, sexual risk behaviour, health and wellbeing.

## Abstract

**Background:**

The annual number of new human immunodeficiency virus (HIV) infections in the United Kingdom among men who have sex with men (MSM) has risen, and remains high among heterosexuals. Increasing HIV transmission among MSM is consistent with evidence of ongoing sexual risk behavior in this group, and targeted prevention strategies are needed for those at risk of acquiring HIV.

**Objective:**

The Attitudes to and Understanding of Risk of Acquisition of HIV (AURAH) study was designed to collect information on HIV negative adults at risk of HIV infection in the United Kingdom, based on the following parameters: physical and mental health, lifestyle, patterns of sexual behaviour, and attitudes to sexual risk.

**Methods:**

Cross-sectional questionnaire study of HIV negative or undiagnosed sexual health clinic attendees in the United Kingdom from 2013-2014.

**Results:**

Of 2630 participants in the AURAH study, 2064 (78%) were in the key subgroups of interest; 580 were black Africans (325 females and 255 males) and 1484 were MSM, with 27 participants belonging to both categories.

**Conclusions:**

The results from AURAH will be a significant resource to understand the attitudes and sexual behaviour of those at risk of acquiring HIV within the United Kingdom. AURAH will inform future prevention efforts and targeted health promotion initiatives in the HIV negative population.

## Introduction

### Background

During 2013, 6000 people were newly diagnosed with human immunodeficiency virus (HIV) in the United Kingdom (UK), and the estimated number of people living with HIV in the United Kingdom was 107,800 by the end of that year [ 1]. Despite no reported rise in the annual number of new diagnoses since 2005 in the overall UK population, there is evidence that HIV incidence is increasing among men who have sex with men (MSM) [ 2], and in 2013 the number of new HIV diagnoses remained high in black African men and women, who constitute two thirds of all heterosexuals living with HIV in the United Kingdom [ 1].

An estimated 38,700 black Africans were living with HIV in the United Kingdom in 2013. Despite a decline in new diagnoses among people born in sub-Saharan Africa, black Africans form the second largest social group affected by HIV in the United Kingdom [ 1]. The combination of high prevalence of HIV in the black African community [ 1] and the high proportion of undiagnosed infection as a consequence of late presentation [ 3] means that the potential for onward transmission of HIV is high within this community. Although the proportion of late HIV diagnoses has declined overall in the last decade, late diagnosis was highest among black African men (69%) and women (57%) in 2013 [ 1]. Research into factors that affect attitudes towards HIV and access to HIV testing and services is important. The African Health and Sex survey in 2013-2014 demonstrated a low level of awareness on HIV prevalence within black African people living in the United Kingdom, and poor knowledge of HIV treatment and care availability [ 4], which may impact on access to HIV testing services and sexual risk behavior. Furthermore, previous research into late presentation among black Africans demonstrated that HIV awareness did not translate into individual perception of risk or use of services, and that major structural barriers such as stigma, confidentiality and migration issues inhibit the uptake of HIV testing and services [ 5].

Evidence of ongoing, and likely increasing, HIV transmission among MSM [ 1, 2, 6- 8] is consistent with evidence of ongoing sexual risk behavior in this group and there is evidence that the prevalence of condomless sex among MSM in the United Kingdom may have changed over the last few decades. In the United Kingdom the extensive research carried out in community-venue and clinic based studies [ 6, 8- 15] has indicated an increase in the prevalence of condomless anal intercourse among MSM during the late 1990s and early 2000s, coincident with the widespread introduction and use of successful combination antiretroviral treatment (ART) for HIV in developed countries. Research from the United States [ 16, 17] and Europe [ 18- 21] also describes an increase in diagnoses of other sexually transmitted infections (STI) over this time. It has been suggested that the increase in condomless sex that occurred in the late 1990s in the Western world may now have plateaued [ 9], and recent data from NATSAL-3 (a large representative survey of sexual behavior in the UK general population from September 2010 to August 2012) described no change in prevalence of condomless sex or risk perception in MSM over the last decade [ 22]. However, the incidence of HIV in MSM in the United Kingdom appears to have increased [ 2]. This increase cannot be explained by changes in HIV testing alone [ 23], but would be compatible with a modest ongoing increase in condomless sex among MSM [ 2].

Sexual transmission risk arises as a result of perceptions and behaviors which may differ depending on the HIV serostatus of individuals. Strategies aimed at reduction of HIV transmission need to address differences in both HIV positive and negative individuals’ perceptions, choices, and behaviors [ 24]. As increasing evidence shows that a suppressed HIV viral load (VL) greatly reduces the risk of onward transmission of HIV to sexual partners [ 25, 26], it is important to consider how this research might have reached and influenced HIV negative persons in different ways to those who are HIV positive. The PARTNER study recently presented transmission estimates of zero in heterosexuals and MSM for condomless sex where the positive partner was on suppressive ART, albeit with a high upper confidence limit in MSM [ 27]. These data may also influence HIV negative persons in understanding and perception of HIV transmission risks. Research from the United States suggests that HIV negative MSM perceive a number of sexual practices with HIV positive MSM on ART as less risky than with HIV positive MSM who are not on ART [ 24]. Furthermore, evidence from Australia has demonstrated that a behavioral response by MSM to the risk of HIV transmission has evolved considerably over time [ 28]. Risk reduction strategies such as using HIV VL to negotiate condom use [ 28], serosorting (using HIV status as a decision-making point in choosing a sexual partner [ 29, 30]), strategic positioning (choosing a different sexual position or practice depending on the serostatus of a partner [ 31]), negotiated safety (choosing not to use condoms with a primary partner and establishing specific rules for sex outside of the primary relationship [ 32]), and withdrawal are now commonly used to reduce the risk of transmitting or acquiring HIV during condomless anal intercourse.

Current data from the United Kingdom that inform on these themes from the perspective of HIV negative MSM and black Africans are limited. In particular, information is needed on HIV testing behavior and preferences, patterns of sexual behavior, prevalence of specific types of condomless sex (to capture potential risk reduction strategies), attitudes to condomless sex with individuals of known and unknown HIV status, and associations with factors such as mental/general health, STI history, and alcohol and drug use. Data from the Attitudes to and Understanding of Risk of Acquisition of HIV (AURAH) study will contribute to an understanding of how knowledge of ART and detectable/undetectable VLs among HIV negative individuals may affect attitudes and perceptions which lead to condomless sex with partners of unknown and/or known HIV status in the United Kingdom.

Uptake and frequency of HIV testing among MSM in the United Kingdom remains inadequate (an estimated 25% never tested [ 2]), as it does in black Africans (an estimated 40% never tested [ 33]). Therefore, improving efforts to expand testing outside sexual health clinics is a priority, and it is a key recommendation from Public Health England to reduce the burden of undiagnosed HIV in these two groups [ 1]. The first self-testing HIV kit featuring a Kitemark (a UK product and service quality certification) was released in the United Kingdom in April 2015 [ 34]. Although the majority of HIV tests are currently conducted in sexual health clinics, emerging evidence suggests that HIV self-testing is highly acceptable to both MSM and black Africans in low and high income settings [ 35, 36]. HIV self-testing may remove some of the barriers around accessing sexual health services that are experienced by black Africans and MSM, and may help to improve access to HIV testing. However, there is a need to assess HIV testing preferences in HIV negative individuals, given the recent expansion of testing options to include HIV self-testing [ 34], and the need to increase HIV testing in the most at-risk populations in the United Kingdom. Results from the AURAH study will seek to inform on HIV testing preferences and acceptability of HIV testing outside of the traditional sexual health clinic setting.

The AURAH study will allow comparison of HIV negative or undiagnosed MSM and black Africans with HIV positive participants from the Antiretrovirals, Sexual Transmission Risk and Attitudes (ASTRA) study [ 37], a previous questionnaire study undertaken in 2011-2012 by the same group. The ASTRA study focused on patients with HIV under care within the United Kingdom, and asked many of the same questions as the AURAH about sexual behavior, attitudes, and health and lifestyle factors. The ASTRA study aimed to assess sexual risk behaviors, beliefs about HIV transmission risk, and attitudes towards the use of early ART in this population [ 37]. Previous studies have illustrated high prevalence rates of depression, anxiety, and drug and alcohol use in MSM [ 38], whilst black and minority ethnic groups in the UK’s general population are also more likely to be diagnosed with mental health problems and experience poor outcomes from treatment than other ethnic groups [ 39, 40]. There is some evidence that depression in MSM is associated with higher levels of condomless sex and higher risk behaviors [ 41] but there is limited data on mental health and wellbeing, and sexual behavior, among MSM or black Africans in the United Kingdom. Data from the AURAH study will allow insight into these issues. Furthermore, comparison between HIV positive and negative individuals in both MSM and black Africans will help to elucidate the specific effect of HIV and HIV treatments on health, wellbeing, and lifestyle among MSM and black Africans.

This paper describes key aspects underlying the AURAH study, including its rationale, design, methods and response rates. A description of the participant characteristics is also outlined. Details of both response rates and participant characteristics may be of use in the comparison to other studies set in sexual health clinics or outpatient settings, and inform future design and planning of subsequent studies. Further publications will address detailed research questions based on the data collected from the participants in the AURAH study.

### Aims and Objectives

The primary aim of the AURAH study was to assess patterns of sexual behavior, and attitudes to sexual risk, among HIV negative adults at risk of HIV infection, and to investigate associations with demographic, socio-economic, health, and lifestyle factors.

### Study Objectives

The detailed objectives of the AURAH study were to assess the following in HIV negative (not known to be HIV positive) sexual health clinic attendees:

1. Levels of recent condomless vaginal or anal sex according to demographic groups (sexuality, ethnicity).
2. Among those who have had condomless sex, the distribution of: number of sexual partners, type of partners, knowledge of HIV status of partners, number of times had condomless sex, type of condomless sex, and reasons for not using condom.
3. Among those having condomless sex with partners of positive or unknown HIV serostatus, the prevalence of risk-reduction measures such as seropositioning.
4. The prevalence of psychological and physical symptoms (ie, depression, anxiety) and lifestyle factors (ie, drug and alcohol use), and whether demographic/social factors, psychological and physical symptoms, quality of life, and lifestyle factors are associated with condomless sex.
5. Beliefs regarding the effect of ART in HIV positive individuals, and undetectable VL, on HIV transmission risk (transmission risk beliefs) and the association of such beliefs with sexual behavior.
6. History of any HIV testing and attitudes to HIV and HIV medications, including awareness of, and any history of, taking post-exposure prophylaxis (PEP) and pre-exposure prophylaxis (PrEP).
7. Attitudes towards testing for HIV in different settings (ie, sexual health clinic, general practitioner, community based testing), type of testing (ie, self-sampling, self-testing) and preferred sample type for HIV self-testing (ie, saliva based or finger-prick sample of blood).

## Methods

### Study Design

AURAH was a cross-sectional self-administered questionnaire study in individuals attending 20 sexual health (Genito-Urinary Medicine) clinics, in 15 clinical centers (National Health Service trusts), across the United Kingdom. The recruitment period was 17 months, commencing June 2013.

### Population and Setting

AURAH was conducted among individuals attending sexual health clinics for routine STI and/or HIV testing. The inclusion criteria were as follows: HIV negative (or undiagnosed) subjects aged 18 years or over, attending for routine STI or HIV testing in sexual health clinics. Individuals not known to be HIV positive at the time of recruitment to AURAH, but testing positive on that (or a subsequent) clinic visit were retained in the AURAH sample.

The 20 clinical centers were situated across England, and details of the locations and clinics are listed in the Acknowledgements section. The sites were selected on the understanding that they could provide access to large numbers of HIV negative patients attending clinics for STI screening and HIV testing, including the key demographic *at risk*subgroups in the United Kingdom (MSM and black Africans). Most clinics were able to provide a mixed demographic of study participants but a few clinics recruited large numbers of one type only. For example, the 56 Dean Street clinic and the Mortimer Market Center recruited a large number of MSM to the study. Similarly, there were other centers that provided a larger number of black African male and female participants for the study, including the Greenway Center, City of Coventry Healthcare Center, and the Sydenham Center, Barking, London.

### Sample Size

The AURAH study adopted a recruitment target of 2000 total sample size, of which 1000 would be MSM, and 1000 heterosexuals, of whom 600 would be black African. After calculations the study would have sufficient power to:

1. Ascertain the proportion of individuals who report that they have had condomless sex in the past 3 months with a partner of unknown or positive HIV status and that one of the reasons for this was “ *I knew there was a risk of acquiring HIV but I am not so concerned about having the disease that it made me want to have sex using a condom*.” This would be calculated as a proportion of all study participants and as a proportion of all participants reporting condomless sex.
2. Ascertain the proportion of individuals who report that they have had condomless sex in the past 3 months with a positive partner who gave a reason as “ *I thought the risks of catching HIV were low because my partner was taking anti-retroviral therapy.*”
3. Compare the prevalence of depression on the Patient Health Questionnaire (PHQ-9) scale [ 42] between HIV positive and HIV negative individuals, separately for HIV negative MSM, heterosexual men and women, and black African men and women.

For objectives (1) and (2), the planned sample size of 1000 MSM would allow estimation of a 5% prevalence (95% CI 3.65-6.35), a 10% prevalence (95% CI 8.65-11.35), and a 20% prevalence (95% CI 17.52-22.48). For the planned sample size of approximately 300 black African men (or women), prevalences of 5% (95% CI 2.55-7.45), 10% (95% CI 6.60-13.4) and 20% (95% CI 15.47-24.53) would be estimated.

For objective (3), given approximately 2250 MSM, 200 black African men and 450 black African women in the ASTRA sample [ 37], and assuming a prevalence of depressive symptoms of 25% among each of these groups, the study would have 80% power (with 5% 2-sided significance level) and absolute difference in prevalence of 4.5% for MSM, 10.0% for black African men, and 8.5% for black African women.

### Recruitment

**Figure 1:**
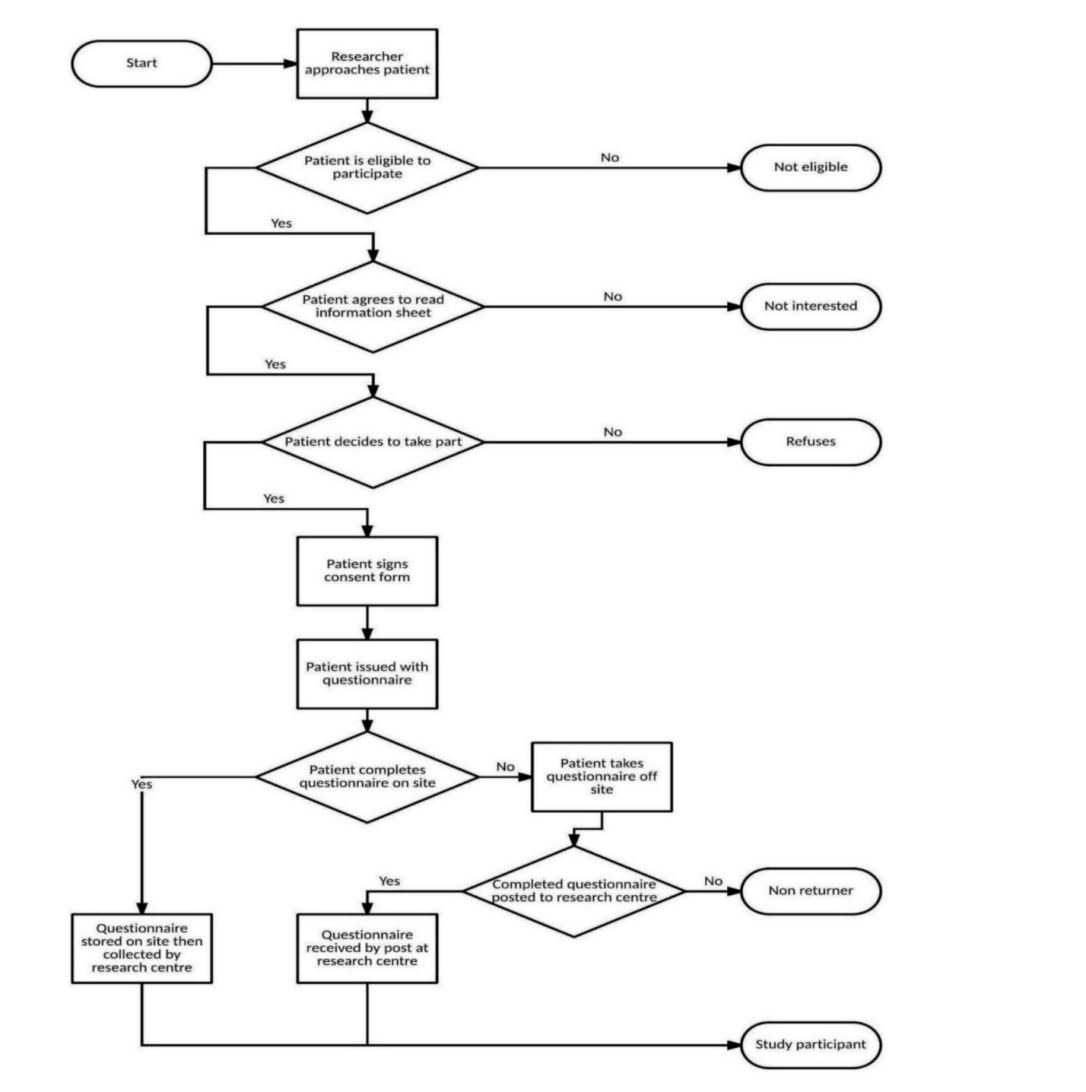
Flowchart of AURAH clinic recruitment.

Recruitment to the study took place between June 2013 and November 2014 during different periods at the 20 clinics. A flowchart of recruitment procedures for the study is included (see Figure 1).

Initial recruitment in the clinics was not restricted. Each site identified specific clinics each week, at which subjects were recruited, aiming to ensure a reasonably representative study population. Consecutive subjects attending each clinic were identified, approached, and invited to take part. It was more feasible to initially recruit in this unrestricted way, and the intent was to modify recruitment strategy as necessary to recruit a sufficient number of MSM and black Africans. After 6 months of unrestricted recruiting, targeted recruitment was implemented across all study sites, and clinic staff were asked to identify and recruit only MSM or those of black ethnicity. Once the recruitment target of 1000 MSM had been met (11 months into the study), 15 clinics were asked to concentrate on recruiting only those known to have specifically black African ethnicity before finishing recruitment, and the 5 sites that had recruited the largest number of MSM continued with sole recruitment of MSM to increase the power for some research questions.

### Consent

All subjects who were invited to participate were given an information sheet about the study. Those who agreed to complete the questionnaire were asked to sign a consent form. The form included an optional section for participants to provide details to allow contact regarding study reminders and, in the future, to invite participants to join future research studies. Participants were informed that consent to be contacted was optional but that those who provided contact details would be entered into a monthly draw offering a prize of £100 of shopping vouchers. Participants who agreed to be contacted were asked for their preferred contact details (email address and mobile phone number for short message service contact). The consent form noted that participants’ contact details would only be used for these purposes and would be held securely at the study management center as part of the study records, but would be deleted after a period of two years. During the consent process, it was reiterated that the study was for HIV negative or undiagnosed individuals only. Participants were told that the questionnaire would take between 15 and 30 minutes to complete and were given an envelope to seal it in, so that their answers were not available to clinic staff. There was an option for participants to take the questionnaire off-site for completion, and postage paid envelopes were provided to return the questionnaire directly to the study management center if required. The option of taking the questionnaire off-site was aimed at including participants who did not have time to complete the questionnaire before they were called for their clinic appointment. Participants were encouraged to complete the questionnaire on-site if possible, to minimize non-return of questionnaires by consented participants.

### Clinical Data

Participants were made aware that their participation included supplying information on the results of any STI or HIV tests that took place in the clinic on the day they were enrolled in to the study. The study log was used to record whether any HIV or STI tests were undertaken and to record the result of any HIV test (negative/positive) performed on the day of enrolment.

### Data Processing

Completed questionnaires were collected in the clinic and transferred regularly to the study management center. Questionnaires were identified only by a unique study number. Participants were instructed not to write their name or clinic number on the questionnaire to maintain their anonymity.

Details of all clinic attendees approached for the study were collected in a study log maintained securely and updated daily at each clinical site. The study log contained study numbers, clinic identifiers and details of consent status for all patients invited to participate in the study, whether or not HIV and other STI tests had been done, and the result of any HIV test. Contact details of participants were also entered in the log if participants consented to being contacted about future research. Selected information from the study log at each clinical center was securely transferred on a regular basis to the study management center. At the study management center, contact details for future research were kept securely and separately from the questionnaire data.

Regular reports were sent from the study management center to each site during the recruitment period, detailing trends and overall progress in recruitment for each of the study sites. In addition, regular checks were made on the completeness and quality of the study log and its concordance with received questionnaires.

Questionnaires received at the management center were digitized by an external data processing contractor. Each paper questionnaire was checked for legibility, digitally scanned, and the resulting images were used as the source for two manual data entry rounds with subsequent quality checking. The completed data entry batches delivered by the contractor were checked for accuracy at the study management center by fully examining a 5% sample.

The original pseudonymized study datasets, including scanned images of the questionnaires, were stored at the study management center in encrypted digital form. They were preserved by being duplicated and stored on managed servers with regular backup and professional administration. The original paper questionnaires were stored securely in locked cabinets. The study datasets will be made freely and readily available to the research community after a suitable interval in a form that ensures that participant anonymity and confidentiality is maintained.

### Study Questionnaire

The questionnaire was based on the design of the ASTRA study questionnaire, a cross sectional study that took place among HIV positive participants attending outpatient HIV clinics across the United Kingdom in 2011-2012 [ 37] that aimed to include a representative sample of outpatients attending for care at each center. The AURAH study questionnaire was adapted to capture relevant information from HIV negative participants. An initial questionnaire design was printed in A5 booklet format and piloted at one study site in June 2013, using the recruitment procedures described above. Following feedback from participants and research staff, minor revisions were made to the final questionnaire, the patient information sheet, the consent form, and an insert was designed to attain further information on preferences for HIV testing. These changes were submitted as amendments for ethical approval and were incorporated into the final version employed during the main recruitment period, which commenced in July 2013.

The final questionnaire consisted of a printed A5 booklet, with versions for men (24-page questionnaire) and women (20-page questionnaire). The pilot study indicated that the questionnaire took roughly 20-25 minutes to complete. The questionnaire sought detailed information on the following factors:

1. *Demographic and social factors*: including gender, age or year of birth, ethnicity, education, employment, housing, financial status, sexuality, relationship status (whether in long-term partnership and HIV-status of partner), country of birth, and number of children.
2. *Health and well-being*: including psychological and physical symptoms (modified version of Memorial Symptom Assessment Scale Short-Form [ 43, 44]), depression (PHQ-9 [ 42]), anxiety (Generalized Anxiety Disorder 7 [ 45]), health-related quality of life (EuroQoL-3L [ 46]), and social support (modified version of the Duke-UNC Functional Social Support Questionnaire [ 47]).
3. *Health and relevant medical history*: including any major medical conditions, recently diagnosed STIs, symptoms of STIs, diagnosed hepatitis B and C, treatment for depression, treatment for other mental health problems, pregnancy status for women, and whether circumcised for men.
4. *HIV-related information*: including HIV status (participants reporting HIV positive status on the questionnaire were excluded from the study), history of any HIV tests, beliefs about transmission risk in relation to ART and undetectable VL, knowledge and history of use of PEP and PrEP, and attitudes towards HIV self-testing and clinic based tests.
5. *Lifestyle factors*: including cigarette smoking status, usual alcohol intake, evidence of alcohol dependency (the CAGE questionnaire [ 48]), recent use of recreational drugs (with details), and recent use of injecting drugs.
6. *Sexual lifestyle*: MSM participants were asked about disclosure of their sexuality to others and involvement in the gay social scene.
7. *Sexual activity*: sexual activity (vaginal or anal sex) during the previous 3 months was ascertained separately for (i) men having sex with women, (ii) men having sex with men, and (iii) women having sex with men. For those participants who reported condomless sex in the past 3 months, there were questions on number of partners, type of partners (long-term or other), attitudes to the risk of HIV infection, and knowledge of the HIV status of partners. There were additional questions on the number and type of partners if the participant reported condomless sex with people known to be HIV positive. All participants were also asked about the use of the Internet to find sexual partners, different sex practices and group sex, attitudes towards disclosure of HIV status to sexual partners and negotiation of condom use, their total number of new sexual partners in the past year, and preferred information sources (if any) about safer sex.
8. *HIV testing preferences*: participants were asked to rank different ways of testing for HIV. Ranking from least liked to most appealing on a scale of 1-4, the options were (i) in a sexual health clinic, (ii) general practitioner, (iii) self-sampling, and (iv) self-testing. Participants were also asked to indicate a preference for saliva or blood based self-testing options.

### Ethics Statement

The research protocol and all versions of the study documents for the AURAH study (information sheet, consent form, questionnaires and insert) were approved by the designated Research Ethics Committees (REC) (National Research Ethics Service committee London-Hampstead, ref: 13/LO/0246). Based on these documents, the study subsequently received permission for clinical research at all participating National Health Service (NHS) sites from local Research & Development (R&D). The REC (NRES committee London-Hampstead) further approved the protocol and study documents for the AURAH2 study in December 2014 (REC ref: 14/LO/1881) and subsequent permission by local R&D for clinical research at the three NHS clinic sites in March 2015.

### Study Management

The study was managed on a day-to-day basis by a core group of five staff at the study management center: the HIV Epidemiology and Biostatistics Group, Research Department of Infection and Population Health, Royal Free Campus, University College London.

An advisory group was also established at the start of the study to provide guidance and support. The advisory group consisted of representatives from University College London, HIV i-Base, the London School of Hygiene and Tropical Medicine and City University London.

## Results

Over the 17-month study period a total of 4393 eligible patients were approached and asked to participate in this study. Of those approached, 3340 (76.03%) gave consent to take part in the study. The number of completed questionnaires finally collected was 2630 and thus the response rate was 59.87% (2630/4393) of eligible patients approached, and 78.74% (2630/3340) of those who gave consent. The majority of respondents (1432/2630, 54.44%) agreed to provide their contact details for participation in future research.

Eighteen of the 20 participating clinics were able to provide estimates of the number of outpatients seen in all clinical sessions over the same period, and the numbers of these in the key groups (MSM and black Africans). More than 288,090 patients were found to have attended these 18 clinics at some point during the respective recruitment periods. Of the combined total attending the clinics, it was estimated that approximately 7.6% were black African and 13.6% were MSM. Table 1shows the patient population (recruited to AURAH) and response rates for the 20 clinical centers.

### Characteristics of Those Recruited

The mean age (of the 2630 participants who supplied details) at the time of questionnaire completion was 32 years (SD 10, range 18-80 years). Overall, 1954 (74.30%) participants were men and 676 (25.70%) were women. Of the 1939 male participants whose sexuality was known, 1484 (76.53%) self-classified as MSM and 455 (23.47%) as heterosexual. Of the 1484 MSM participants, 965 (65.03%) agreed to provide their contact details for participation in future research, whereas only 36.92% (168/455) of heterosexual males and 43.20% (292/676) of females agreed to provide these details.

In terms of ethnic origin, 1505 of the 2630 (57.22%) participants self-classified as white, 580 participants (22.05%) as black African ethnicity, 249 (9.47%) as other black ethnicity, 264 (10.04%) as other ethnicity, and ethnic status was missing for 32 (1.22%). Of 548 people of black African ethnicity, 323 (58.9%) were female and 225 (41.1%) were male. Of 250 men of black African ethnicity whose sexuality was known, 30 (12.0%) self-classified as MSM and 220 (88.0%) as heterosexual. Of the 580 participants of black African ethnicity, 213 (36.7%) agreed to provide contact details for participation in future research.

Overall, 2535 of the 3340 consenting participants (75.90%) took an HIV test on the day they were approached in clinic. Of those tested, 18 of 2535 (0.71%) received a positive result that they were unaware of at the time. Of these 18 participants, nine returned completed questionnaires (these are retained in the AURAH sample). All nine of these cases were male, of which five were MSM and four were black heterosexuals. Clinics reported that 2624 of the 3340 consenting (78.56%) also tested for STIs on the day, although information on the nature of each test and the results were not collected for this study.

The characteristics of those recruited at the 20 clinical centers in terms of gender, sexual orientation, relevant ethnic status and testing are detailed in Table 2.

**Table 1:** Recruitment results for the 20 AURAH study clinical centers, 2013-2014.

Site	Length of study period in days	Individual patients attending during recruitment period	Eligible patients approached	Patients consenting (as % of approached = consent rate)	Patients responding = completed questionnaires received (as % of approached = response rate)
Barking	335	3475	64	59 (92%)	34 (53%)
Barts	31	^a^	16	13 (81%)	11 (69%)
Birmingham	127	^a^	53	49 (92%)	33 (62%)
Brighton	482	13918 ^c^	243	240 (99%)	227 (93%)
Bristol	312	1021	59	58 (98%)	55 (93%)
Calderdale & Huddersfield	428	13662	92	82 (89%)	73 (79%)
Coventry	337	11218	269 ^b^	256 (95%)	246 (91%)
Dean Street	473	51882 ^d^	1384	895 (65%)	604 (44%)
Homerton	300	25312	159	149 (94%)	123 (77%)
John Hunter	450	20236 ^d^	235	131 (56%)	84 (36%)
Kings	283	15500	305	204 (67%)	168 (55%)
Leicester	84	5173	69	66 (96%)	48 (70%)
Mortimer Market	332	13652 ^e^	382	370 (97%)	313 (82%)
Newham	320	9203	168	119 (71%)	113 (67%)
Reading	405	14807	82	75 (91%)	75 (91%)
Royal Free	416	33216	137	126 (92%)	101 (74%)
St George's	333	17041	110	90 (82%)	81 (74%)
The London	247	13747	40	35 (88%)	33 (83%)
WLCSH	463	19094 ^d^	462	270 (58%)	164 (35%)
Whipps Cross	314	5933	64	53 (83%)	44 (69%)
TOTALS	-	288090	4393	3340 (76%)	2630 (60%)

^a^Clinic unable to supply data on total clinic attendance

^b^Clinic was unable to supply data about those declining to participate – value derived from 95% consent rate estimated by the clinic

^c^Covers 75% of the recruitment period only

^d^Covers 90% of the recruitment period only

^e^Covers 55% of the recruitment period only

**Table:** 

Site label	Men (as % of questionnaires received)	MSM (as % of questionnaires received)	Black African men (as % of questionnaires received)	Women (as % of questionnaires received)	Black African women (as % of questionnaires received)	Tested for HIV on the day (as % of consenting)	STI test on the day (as % of consenting)
Barking	25 (74%)	8 (24%)	16 (47%)	9 (26%)	9 (26%)	43 (73%)	49 (83%)
Barts	11 (100%)	11 (100%)	0 (0%)	0 (0%)	0 (0%)	13 (100%)	13 (100%)
Birmingham	11 (33%)	7 (21%)	0 (0%)	22 (67%)	3 (9%)	40 (82%)	49 (100%)
Brighton	207 (91%)	197 (87%)	5 (2%)	20 (9%)	9 (4%)	170 (71%)	185 (77%)
Bristol	51 (93%)	44 (80%)	2 (4%)	4 (7%)	3 (5%)	55 (95%)	38 (66%)
Calderdale & Huddersfield	55 (75%)	47 (64%)	6 (8%)	18 (25%)	11 (15%)	69 (84%)	71 (87%)
Coventry	104 (42%)	34 (14%)	57 (23%)	142 (58%)	105 (43%)	124 (48%)	161 (63%)
Dean Street	585 (97%)	528 (87%)	14 (2%)	19 (3%)	7 (1%)	762 (85%)	618 (69%)
Homerton	70 (57%)	37 (30%)	8 (7%)	53 (43%)	19 (15%)	88 (59%)	122 (82%)
John Hunter	69 (82%)	49 (58%)	4 (5%)	15 (18%)	1 (1%)	94 (72%)	98 (75%)
Kings	65 (39%)	31 (18%)	13 (8%)	103 (61%)	25 (15%)	136 (67%)	164 (80%)
Leicester	14 (30%)	8 (17%)	2 (4%)	34 (71%)	11 (23%)	50 (76%)	54 (82%)
Mortimer Market	303 (97%)	266 (85%)	28 (9%)	10 (3%)	7 (2%)	323 (87%)	328 (89%)
Newham	61 (54%)	7 (6%)	50 (44%)	52 (46%)	41 (36%)	98 (82%)	113 (95%)
Reading	50 (67%)	35 (47%)	8 (11%)	25 (33%)	16 (21%)	64 (85%)	68 (91%)
Royal Free	41 (41%)	29 (29%)	6 (6%)	60 (59%)	16 (16%)	98 (78%)	116 (92%)
St George's	56 (69%)	36 (44%)	10 (12%)	25 (31%)	7 (9%)	65 (72%)	82 (91%)
The London	17 (52%)	10 (30%)	5 (15%)	16 (48%)	14 (42%)	26 (74%)	35 (100%)
WLCSH	134 (82%)	92 (56%)	7 (4%)	30 (18%)	3 (2%)	169 (63%)	210 (78%)
Whipps Cross	25 (57%)	8 (18%)	14 (32%)	19 (43%)	18 (41%)	48 (91%)	50 (94%)
TOTALS	1954 (74%)	1484 (56%)	255 (10%)	676 (26%)	325 (12%)	2535 (76%)	2624 (79%)

## Discussion

The AURAH study recruited 2630 participants from 20 UK sexual health clinics during 2013-2014. The initial rate for consent (2630/3340, 78.74%) was relatively high in this study, and the overall response rate (questionnaires received) was 59.87% (2630/4393) of eligible patients approached. However, there was considerable variation between the clinics in the response rate achieved (ranging from 35% to 93%). The difference in response rates between the clinics could be due to a number of reasons. When researchers at the sites with low response rates were asked about potential barriers to participation they noted education and literacy levels, level of English fluency, and the perceived amount of time that the study questionnaire would take to complete, among clinic attendees at their sites. It was felt that the monthly prize draw had not had a significant effect as an incentive to participate but potentially a smaller cash sum might have, however the study did not seek ethical approval for this due to time restraints.

The intention of this study was also to recruit large numbers within the key demographic sub-groups most affected by HIV in the United Kingdom, namely MSM and black African men and women. The study succeeded in this aim, and there were 2034 individuals in these groups of interest: 1484 MSM participants and 580 black African participants, with 30 individuals (1.47%) falling into both of these categories.

It is difficult to compare the overall study response rate with other studies of HIV negative MSM, as many Internet or venue-based studies have no records of numbers not agreeing to participate, and therefore response rate cannot be calculated. Our response rate is comparable with other surveys taking place outside the clinical context that have investigated sexual behavior (70% [ 49]; 65% [ 50]), and with the previous ASTRA study on HIV positive patients whose response rate was 64% [ 37]. Many of the 1746 non-responding eligible patients were those who directly refused to participate (1036/1746, 59.34%). However, the remaining 710 were consenting participants who took a questionnaire away but did not return it (710/1746, 40.66% of non-responders). Although the option of taking a questionnaire off-site for completion was intended to maximize participation, some of the non-response in this study can be attributed to factors impacting upon questionnaire completion and postage after the questionnaires had been taken away from the study site. For example, lack of time or continued motivation to complete and post the questionnaire. However, overall the consent rate was slightly lower than in the comparable ASTRA study and this may reflect the differences between the respective clinic populations in terms of potential ongoing engagement with care, and familiarity with the clinic research staff among those attending HIV and general sexual health clinics.

The average age of AURAH study participants was 32 years and, as expected, this was much younger than the average (45 years) of the ASTRA (HIV diagnosed) study participants [ 37]. The lower mean age for AURAH is consistent with the study’s intention to sample from large numbers of currently HIV negative but *at risk*individuals, who could be expected to be younger than the HIV positive population.

The study population was not a random sample of those attending the clinics, as targeted recruitment was implemented after 6 months of recruitment. It should be noted that the target number for MSM recruitment (1000) was exceeded and that the target was reached early in the study. Recruitment was continued because it was desirable to increase power for some research questions. The number of black Africans recruited was 548, however this took a long time to achieve and required selective recruitment in 15 centers. A similar pattern of relative difficulty of recruitment in these two respective populations was observed in the ASTRA study (on HIV diagnosed individuals) [ 37] where it was found that MSM were over-represented in its sample in relation to the national HIV positive population, and conversely black Africans were under-represented. However, the relative difficulty of recruitment in AURAH may also be a reflection of the different proportions within the populations attending the sexual health clinics, with the overall proportion of MSM (13.6%) being almost double the proportion of black Africans (7.6%).

The number of study participants diagnosed as HIV positive in-clinic during this study was 18 (0.71%, 95% CI 0.38-1.04, of 2535 consenting and tested). The selective nature of our sampling means this is not a meaningful prevalence estimate but, as might be expected in those attending sexual health clinics, this is very much higher than the general UK population HIV estimate for undiagnosed HIV of 0.07% [ 1]. However, it should be noted that in those identifying as MSM, there were 5 HIV positive cases out of 1484 (0.34%, 95% CI 0.04-0.63) in this study, which is a relatively low value when compared with the estimated 0.94% for the prevalence of undiagnosed HIV in the UK MSM population as a whole [ 1]. This could reflect the fact that MSM who regularly attend STI clinics are likely to test more frequently for HIV than MSM in the United Kingdom overall.

### Conclusions

In summary, the AURAH study includes a substantive but selective sample of those considered to be at risk of being infected with HIV in the United Kingdom. AURAH will give insights into the relationships between socio-demographic factors, physical and psychological symptoms, lifestyle factors, health-related quality of life, and sexual behavior in this population.

The results of the AURAH study will be relevant for understanding the process of HIV transmission within the United Kingdom, and for targeting of national prevention efforts. The data from AURAH will contribute to understanding the social, psychological and health-related factors that are linked to high risk sexual and HIV-testing behaviors, and therefore to ongoing transmission of HIV in the two most *at risk*groups of people in the United Kingdom.

## Abbreviations

ART: antiretroviral treatment
ASTRA: Antiretrovirals, Sexual Transmission Risk and Attitudes
AURAH: Attitudes to and Understanding of Risk of Acquisition of HIV
HIV: human immunodeficiency virus
MSM: men who have sex with men
NHS: National Health Service
NRES: National Research Ethics Service
PEP: post-exposure prophylaxis
PHQ: Patient Health Questionnaire
PrEP: pre-exposure prophylaxis
R&D: Research and Development
REC: Research Ethics Committee
STI: sexually transmitted infection
UK: United Kingdom
VL: viral load
